# HVDROPDB datasets for research in retinopathy of prematurity

**DOI:** 10.1016/j.dib.2023.109839

**Published:** 2023-11-25

**Authors:** Ranjana Agrawal, Rahee Walambe, Ketan Kotecha, Anita Gaikwad, Col. Madan Deshpande, Sucheta Kulkarni

**Affiliations:** a Dr. VishwanathKarad MIT World Peace University, Pune, India; b Symbiosis Centre for Applied AI, Symbiosis International (Deemed) University, Pune, India; c PBMA's H. V. Desai Eye Hospital, Pune, India

**Keywords:** Retinopathy of prematurity, Dataset, HVDROPDB, Segmentation

## Abstract

Retinopathy of prematurity (ROP) is a retinal disorder that may bring about blindness in preterm infants. Early detection and treatment of ROP can prevent this blindness. The gold standard technique for ROP screening is indirect ophthalmoscopy performed by ophthalmologists. The scarcity of medical professionals and inter-observer heterogeneity in ROP grading are two of the screening concerns. Researchers employ artificial intelligence (AI) driven ROP screening systems to assist medical experts. A major hurdle in developing these systems is the unavailability of annotated data sets of fundus images.

Anatomical landmarks in the retina, such as the optic disc, macula, blood vessels, and ridge, are used to identify ROP characteristics. HVDROPDB is the first dataset to be published for the retinal structure segmentation of fundus images of preterm infants. It is prepared from two diverse imaging systems on the Indian population for segmenting the lesions mentioned above and annotated by a group of ROP experts. Each dataset contains retinal fundus images of premature infants with the ground truths prepared manually to assist researchers in developing explainable automated screening systems.

Specifications Table

**Table:** 

Subject	Ophthalmology
Specific subject area	Retinal image analysis for ROP
Type of data	Image
How the data were acquired	The technicians captured the fundus images using two wide-angle field imaging systems: RetCam and Neo.
Data format	Analyzed
Description of data collection	The subjects had a gestation age of 26-36 weeks, and their birth weight ranged from 760-3000g. An eye drop was prepared using tropicamide and phenylepinephrine to dilate the patient's pupil before taking the photos. The images were cliqued by the posterior, temporal, superior, inferior, and nasal views of the left and right eyes of the infant using RetCam and Neo. There were approximately 2 to 12 images clicked per eye.
Data source location	PBMA's H.V. Desai Eye Hospital, Pune
Data accessibility	Repository name: Mendeley Data Data identification number: Reserved DOI: 10.17632/xw5xc7xrmp.1 Direct URL to data: https://data.mendeley.com/datasets/xw5xc7xrmp/2
Related research article	Agrawal R, Kulkarni S, Walambe R, Kotecha K. Assistive Framework for Automatic Detection of All the Zones in Retinopathy of Prematurity Using Deep Learning. J Digit Imaging. 2021 Aug;34(4):932-947. https://doi.org/10.1007/s10278-021-00477-8 Agrawal, R., Kulkarni, S., Walambe, R. et al. Deep dive in retinal fundus image segmentation using deep learning for retinopathy of prematurity. Multimed Tools Appl (2022). https://doi.org/10.1007/s11042-022-12396-z

## 1 Value of the Data

• ROP may cause blindness in preterm infants. Preterm births are increasing due to improved neonatal intensive care, and the burden of ROP is expected to rise dramatically. Unfortunately, the ophthalmologists-to-patient ratio is very low and different experts' diagnoses are not unanimous. AI-based automated screening systems are needed to assist clinicians in ROP screening. ROP datasets are not published.
• This dataset provides annotated fundus images of premature infants acquired by two imaging systems, RetCam and Neo. The ground truths (masks) of fundus images are prepared manually with Adobe Photoshop to segment the optic disc, vessels, and demarcation line/ridge. The researchers can use these data to segment the retinal structure essential for detecting zones and stages and develop explainable automated ROP screening systems.
• A framework has been developed for automatically detecting and explaining zones, plus, and stages in the fundus images of infants.

## 2 Objective

Retinopathy of prematurity (ROP) is a disease that affects the retina of a premature infant. It usually affects both eyes and can result in lifelong vision impairment or blindness. ROP blindness is increasing due to improving neonatal intensive care in low and middle-income countries[1]. ROP may progress or regress after a few weeks of the infant's birth. Timely screening is necessary to control ROP progress because if the disease progresses to stage 3 with plus disease, invasive procedures may be required to stop further retinal detachment [2]. Due to the scarcity of medical experts, researchers are developing automated screening systems to assist the experts. The lack of annotated public datasets is a major issue in designing and explaining such systems [3,4].

The following characteristics define the severity of ROP: blood vessel growth by zones (disease location), stages (severity of abnormal growth) seen, a plus disease (vessel size and tortuosity) observed, and the extent (number of clock hours involved) of the disease [5]. This work aims to provide an ROP dataset for segmenting demarcation line/ ridge, optic disc, and vessel for creating an explainable ROP diagnosis system.

## 3 Data Description

**Fig. 1:**
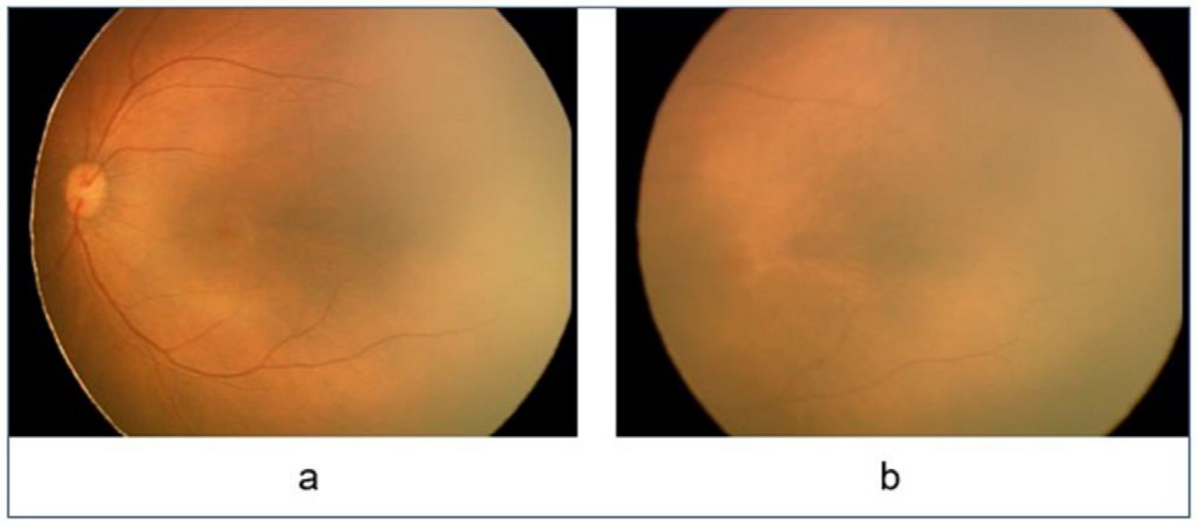
RetCam images (a) posterior view, (b) temporal view.

**Fig. 2:**
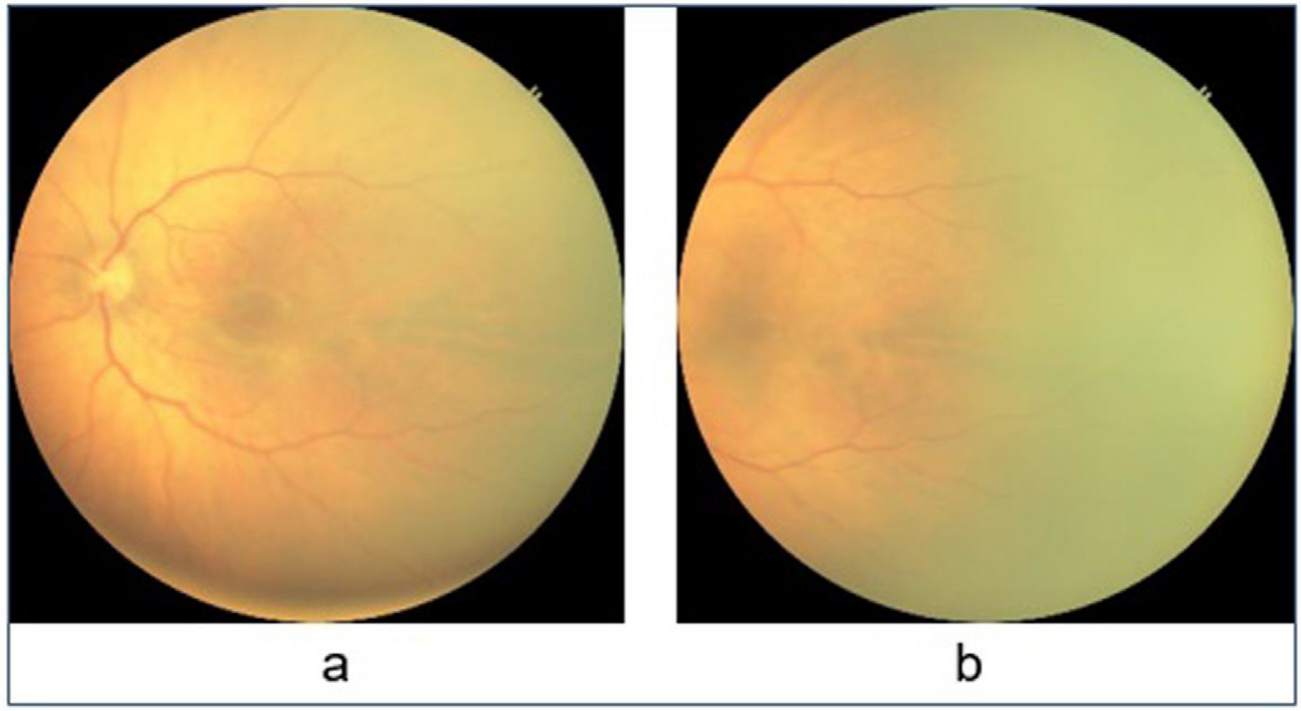
Neo images (a) posterior view, (b) temporal view.

**Fig. 3:**
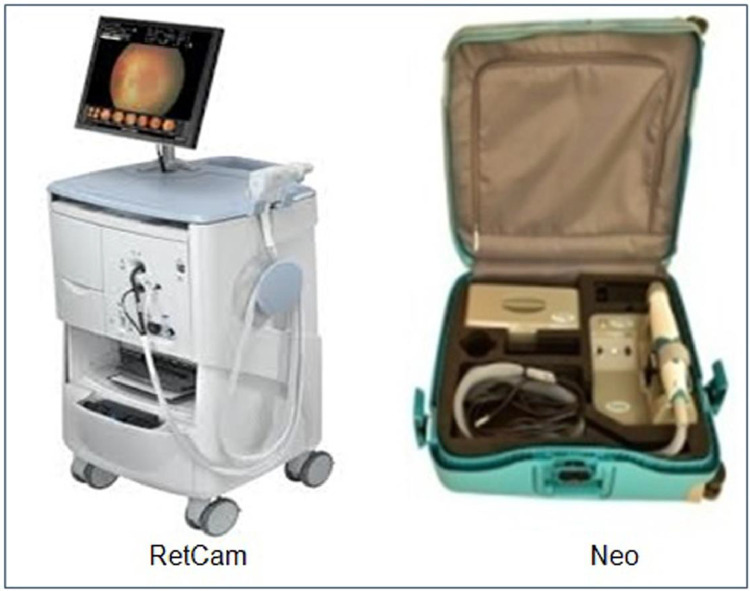
RetCam and Neo imaging systems.

The HVDROPDB dataset consists of posterior and temporal view fundus images of premature infants, as shown in Figs. 1 and 2. Figs. 1a and 2a display posterior images and Figs. 1b and 2b depict temporal images. HVDROPDB was named after the H.V. Desai Eye Hospital in Pune, India, where the fundus images of premature infants were collected. These images were captured by RetCam(Clarity MSI, US) and Neo(Forus Healthcare, Bangalore, India) imaging systems shown in Fig. 3. RetCam is used worldwide. Neo is very popular in India as it is reasonably priced and portable. The RetCam and Neo images are provided separately in these datasets.

HVDROPDB-RetCam-Neo-Segmentation is the first dataset to be published to segment ROP images. It aims to aid in researching automated ROP screening systems and their explanation. The fundus images and their ground truths will facilitate the segmentation of retinal structures essential for detecting zones and stages.

**Fig. 4:**
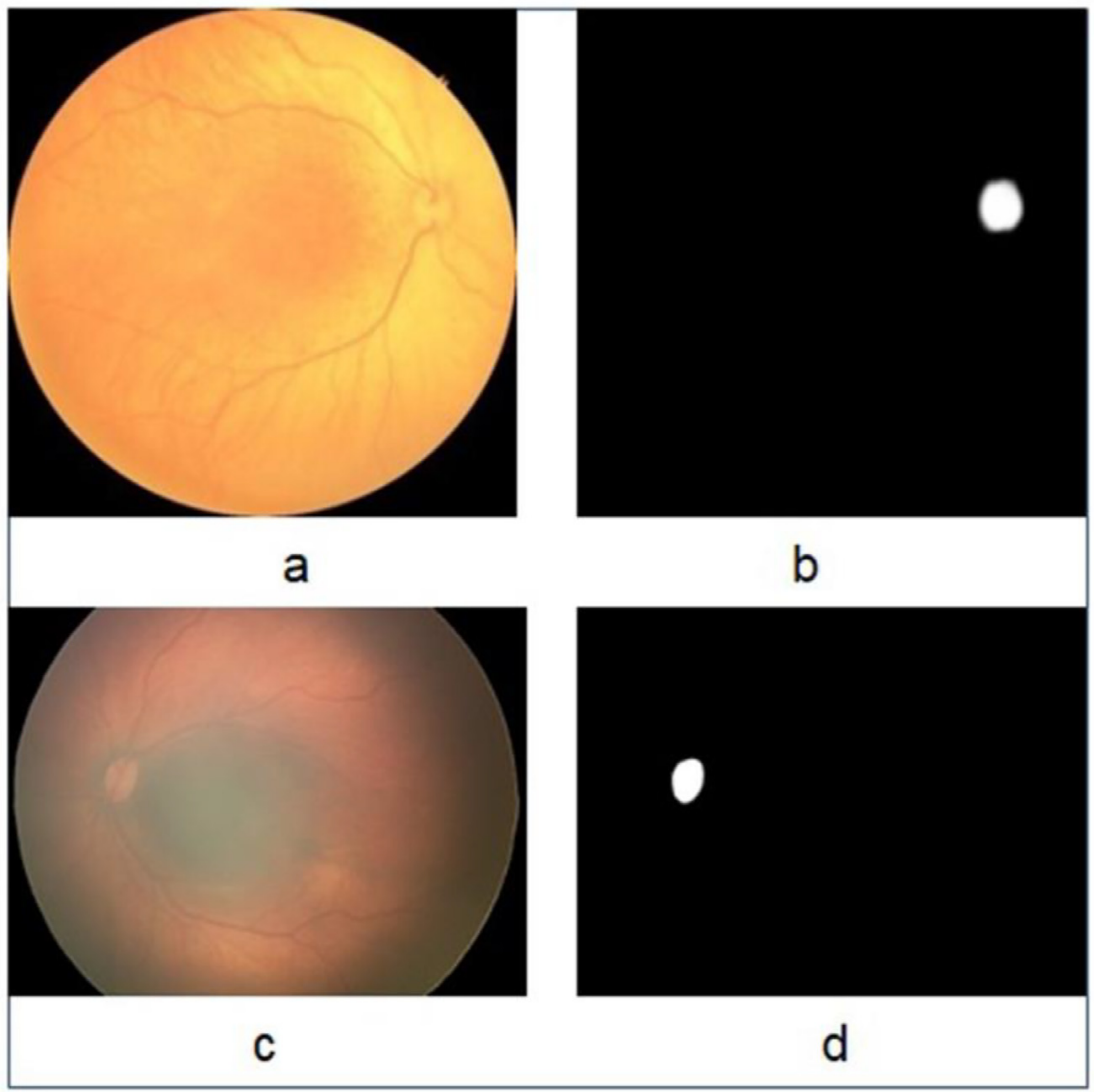
Original images and masks for Optic disc segmentation: (a) Neo image, (b) Neo mask, (c) RetCamimage, and (d) RetCammask.

HVDROPDB_RetCam_Neo_Segmentation dataset was prepared with three primary datasets of HVDROPDB-OD, HVDROPDB-BV, and HVDROPDB-RIDGE for the optic disc, blood vessels, and demarcation line/ridge segmentation, respectively. Each dataset contained four sub-datasets of 50 images and their masks (ground truths), as described in Table 1. An optic disc is seen in the images taken from a posterior view. HVDROPDB-OD dataset was prepared with posterior view images, and it contains two subsets, RetCam_OpticDisc_images and RetCam_OpticDisc_masks, which were used to segment the optic discs in RetCam images. In addition, Neo_OpticDisc_images and Neo_OpticDisc_masks were also included for segmenting optic discs in Neo images. The masks for segmentation were manually created using Adobe Photoshop Reader, as shown in Fig. 4.

**Table 1:** HVDROPDB_RetCam_Neo_Segmentation dataset description.

Dataset name	Sub-dataset	Application	Number of images
HVDROPDB-OD	RetCam_OpticDisc_images	Optic disc segmentation	50
	RetCam_OpticDisc_masks		50
	Neo_OpticDisc_images		50
	Neo_OpticDisc_masks		50
HVDROPDB-BV	RetCam_OpticDisc_images	Blood vessels segmentation	50
	RetCam_OpticDisc_masks		50
	Neo_OpticDisc_images		50
	Neo_OpticDisc_masks		50
HVDROPDB-RIDGE	RetCam_OpticDisc_images	Demarcation line/ridge segmentation	50
	RetCam_OpticDisc_masks		50
	Neo_OpticDisc_images		50
	Neo_OpticDisc_masks		50

**Fig. 5:**
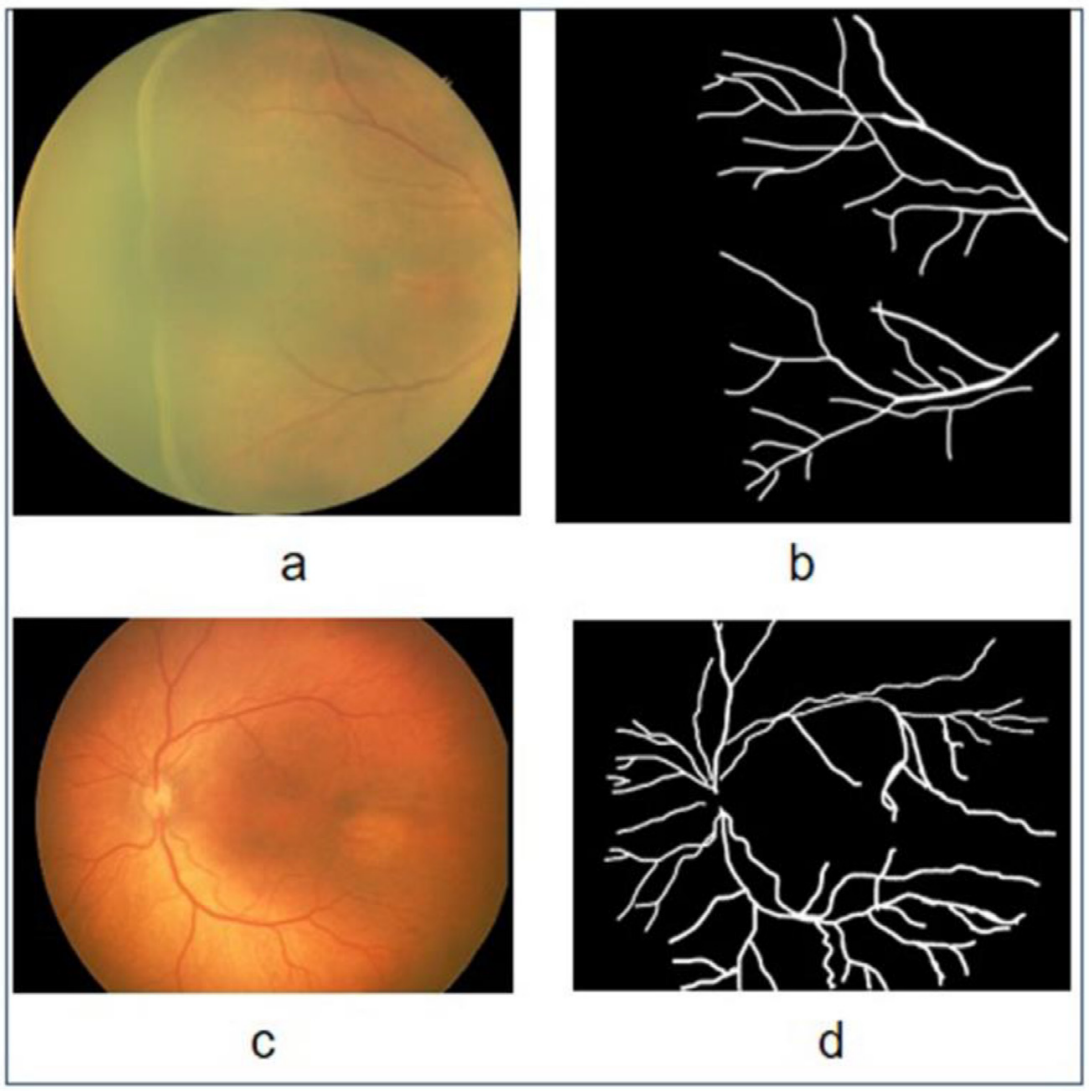
Original images and masks for vessel segmentation: (a) Neo image, (b) Neo mask, (c) RetCamimage, and (d) RetCam mask.

For the creation of HVDROPDB-BV, 100 images captured from the temporal and posterior views were selected, and their ground truths were prepared as shown in Fig. 5. HVDROPDB-BV held RetCam_Vessels_images, RetCam_Vessels_masks, Neo_Vessels_images, and Neo_Vessels_masks datasets each with 50 images.

**Fig. 6:**
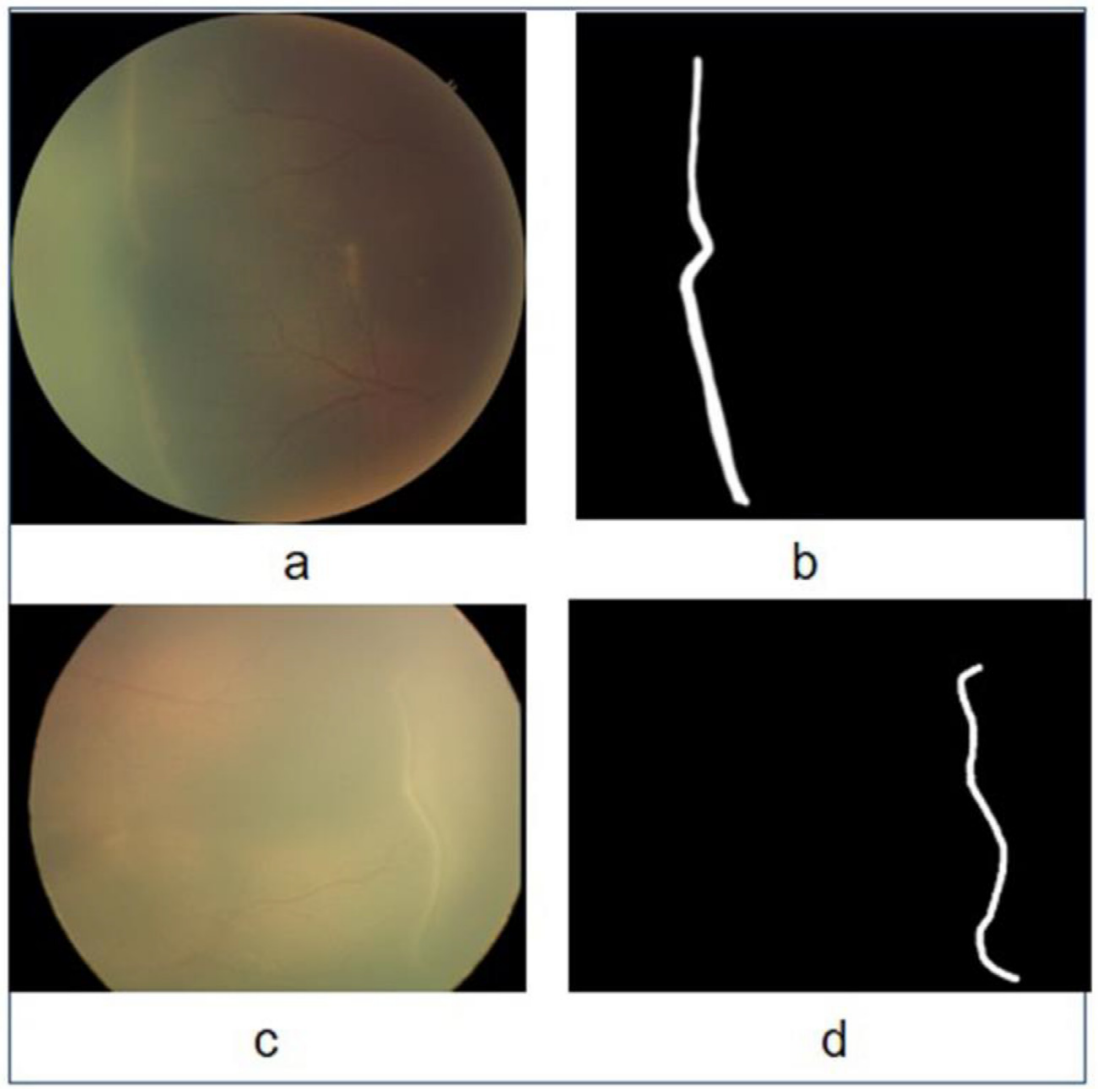
Original images and masks for demarcation line/ridge segmentation: (a) Neo image, (b) Neo mask, (c) RetCamimage, and (d) RetCam mask.

HVDROPDB-RIDGE contained 100 images of ROP stages 1, 2, and 3 captured from both posterior and temporal views, along with their ground truths depicted in Fig. 6. The dataset was divided into four sub-datasets such as RetCam_Ridge_images, RetCam_Ridge_masks, Neo_Ridge_images, and Neo_Ridge_masks. Therefore, a total of 12 datasets were provided for segmentation.

## 4 Experimental Design, Materials, and Methods

**Fig. 7:**
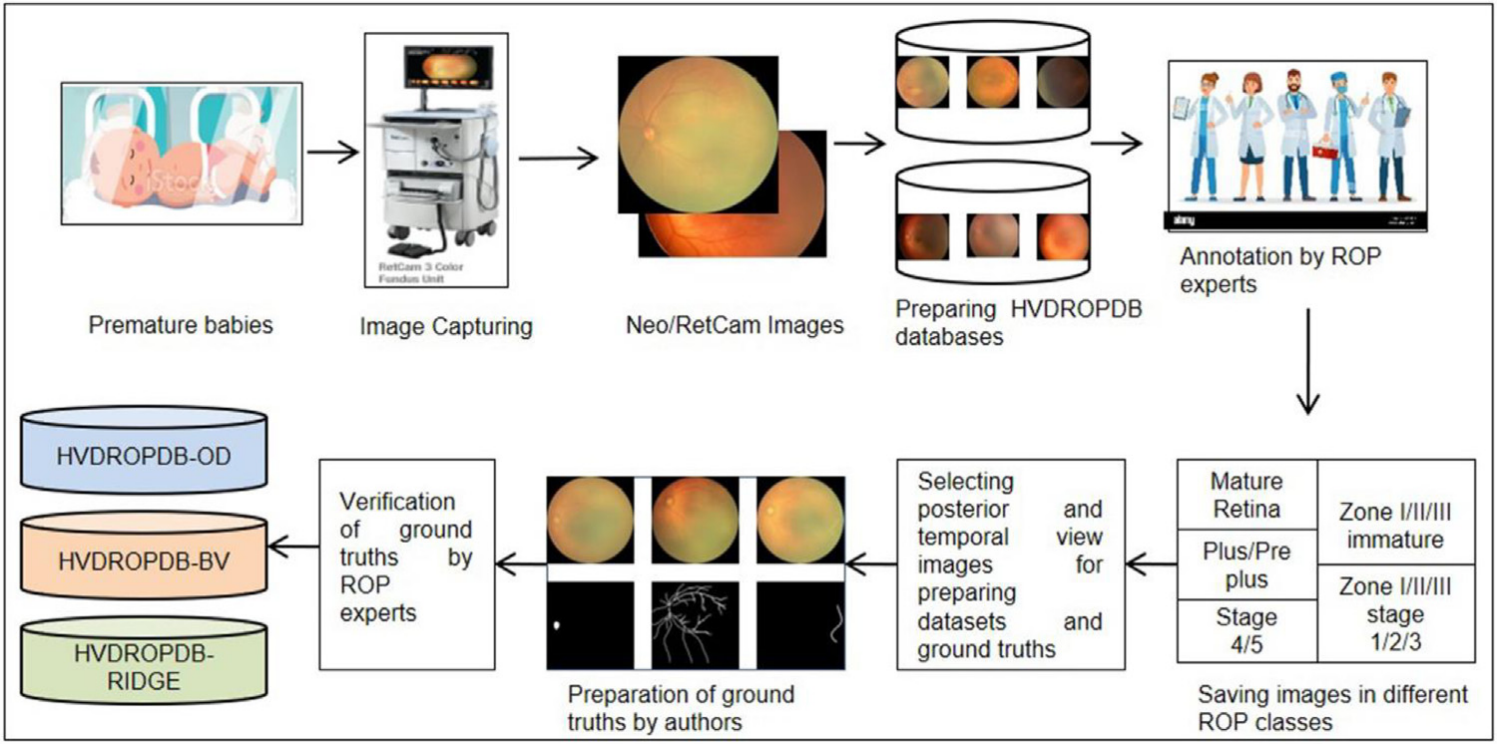
The dataset preparation process.

The dataset preparation process is depicted in Fig. 7. Images were provided by PBMA's H. V. Desai Eye Hospital in Pune captured between the years 2009 and 2022. The subjects were premature infants screened for ROP by the hospital team. The images were obtained by trained optometrists using two Neo or Retcam cameras with 120^◦^ field of view (FOV). Posterior and temporal view images were saved in the database. A team of ROP experts with a minimum of 5 years of experience annotated them under the guidance of a senior ROP expert with 25 years of experience. Before annotation, an interobserver variability test was carried out (Kappa value 0.92). However, possibility of subjective bias cannot be ruled out as there was no external expert involved in annotation. The images were saved as different ROP classes in the HVDROPDB dataset.

### 4.1 Data acquisition

The subjects were 26–36 weeks gestation and weighed 3000 g or less. Their personal information was kept confidential with the hospital, and written informed consent was obtained from their parents for using the data for research purposes. A readymade eye drop (especially diluted Phenylephrine and tropicamide drops) was used to dilate pupils before image acquisition. This eye drop was put three times in each eye at the interval of 10 min. The feeding of the baby was stopped after the third drop.

The posterior, temporal, superior, inferior, and nasal view images were acquired of each eye of the infant using RetCam and Neo. Approximately 2 to 12 images were acquired per eye, yielding 10,570 RetCam and 8280 Neo images from 1100 patients. RetCam images had a resolution of 640×480 pixels and were stored in .png format, whereas Neo images had a resolution of 2040×2040 pixels and were stored in .jpeg format. The size of the RetCam image is approximately 623 KB, whereas the Neo image is 223 KB.

### 4.2 Annotation of images

All collected fundus images of premature infants were gathered in a database. A team of medical experts who are experienced in grading ROP images for telemedicine models labelled these images. Each expert was trained to standardize the annotation process to develop an AI algorithm. Two hours per week were allotted for annotation. The authors reviewed the available literature and discussed it with the ROP experts. As the temporal and posterior views of the images were sufficient for the diagnosis, the team selected a pair of images with these views for each eye. Images of laser-treated infants and those with retinal detachment (a complication of ROP) were excluded. Around 1900, Retcam and 1100 Neo images were stored separately in RetCam and Neo databases. The number of Normal images was much more than the ROP images. To make the dataset robust, poor-quality images were not removed. We are providing here a few images as the ground truth preparation is a complex task, and our research and data collection is in progress.

**Fig. 8:**
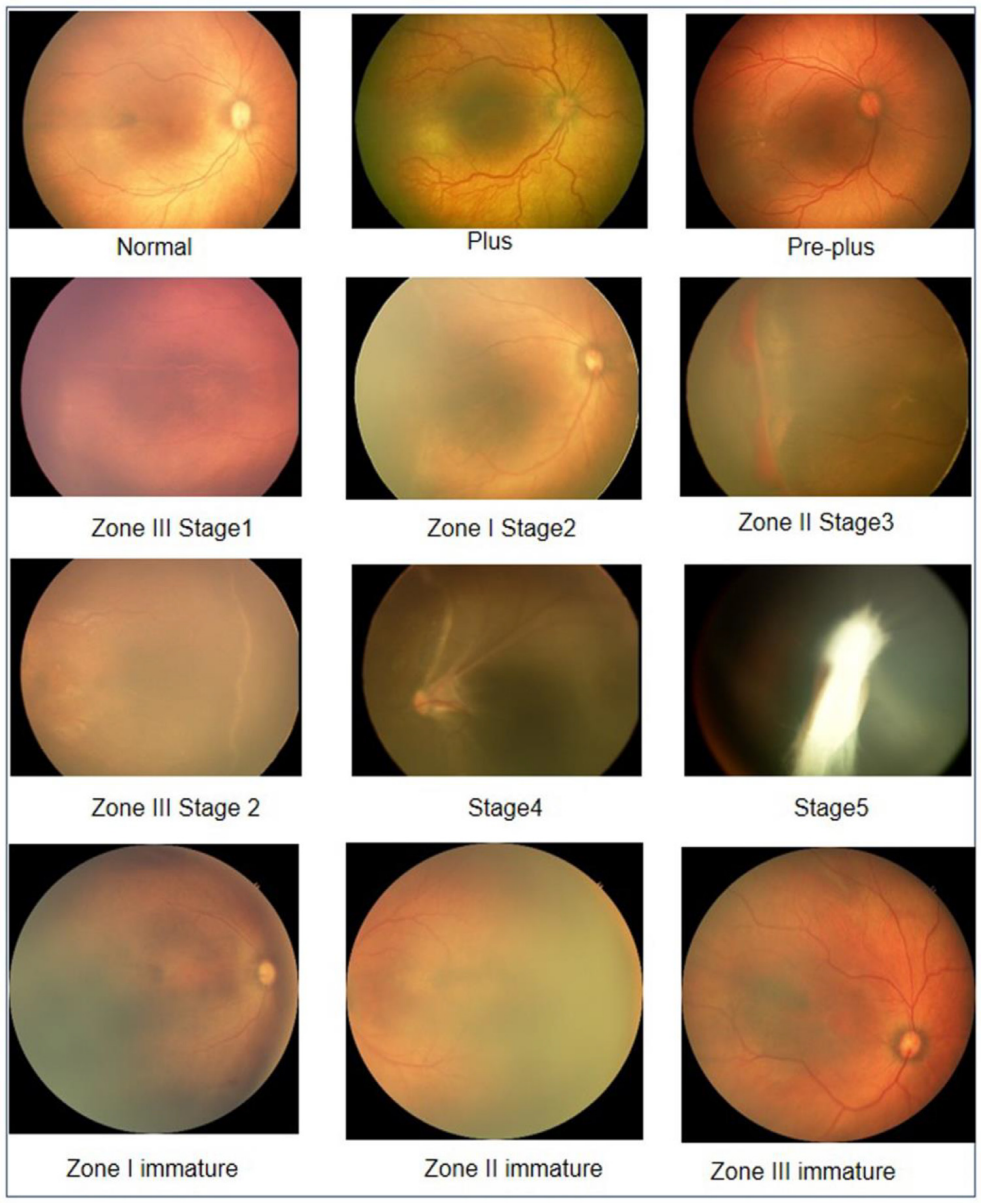
ROP and No-ROP images.

Based on the lesions or the normal structures and the quadrant in a particular image, the ROP expert team has assigned a reference standard diagnosis (RSD). They explained the procedure of diagnosis to the author while labelling the images. Senior ROP expert corrected or confirmed the diagnosis when there was a difference in the assessment. According to the ICROP, each pair of pictures (temporal and posterior) was classified into the following ROP classes: Mature retina, Zone I/II/III immature, Zone I stage 1/2/3, Zone II stage 1/2/3, Zone III stage 1/2/3, Stage 4/5, and Aggressive-ROP. Some of these images are shown in Fig. 8. The Mature retina and Zone I/II/III immature images belong to the No-ROP class. These images were more common than the other mentioned (ROP) classes. Images are unavailable for certain classes, such as “Zone I Stage 3”.

Currently, the research focus is on retinal structure segmentation for ROP explanation. Pixel-level labelling is needed for the optic disc, vessels, and demarcation line/ridge to identify zones and stages of ROP. Hence, the HVDROPDB_RetCam_Neo_Segmentation datasets were prepared to segment the retinal structure.

ROP stages 1, 2, and 3 of posterior and temporal view were gathered from the annotated dataset for HVDROPDB-RIDGE preparation. Similarly, dataset HVDROPDB-OD was prepared with all posterior view images, and HVDROPDB-BV was a combination of both posterior and temporal view images covering most ROP classes. ROP experts explained the retinal structure to the author and the observer in HVDROPDB-OD, HVDROPDB-BV, and HVDROPDB-RIDGE datasets and manually marked the region of interest for segmentation. Ground truths were prepared by the author and the observer using Adobe Photoshop software, and the ROP professionals finally approved them. It is a difficult and time-consuming task that requires continual effort. We have developed a framework on the datasets mentioned above to segment the retina's vascular structure by mixing the RetCam and Neo images with a few additional images and detected zones and stages of ROP [6,7]. The segmentation metrics of the HVDROPDB datasets provided for this study, utilizing the AG U-Net approach [7] on images from Neo and RetCam, are depicted in Table 2 and Table 3. The datasets Neo_OpticDisc_images and Neo_OpticDisc_masks from the HVDROPDB-OD were used for optic disc segmentation of Neo images. The RetCam_OpticDisc_images and RetCam_OpticDisc_masks datasets from HVDROPDB-OD were used for the optic disc segmentation of RetCam images. Similarly, the datasets HVDROPDB-BV and HVDROPDB-RIDGE were used for vessel and ridge segmentation. All datasets were split into training, validation, and testing sets in the ratio 70:10:20 with random state 42.

**Table 2:** Segmentation metrics of HVDROPDB datasets with Neo Images.

Evaluation Metrics	HVDROPDB-OD	HVDROPDB-BV	HVDROPDB-RIDGE
Accuracy	0.99	0.91	0.98
Precision	0.94	0.78	0.75
TPR	0.90	0.57	0.70
TNR	0.99	0.97	0.99
FPR	0.00	0.02	0.00
FNR	0.09	0.42	0.29
Dice Score	0.92	0.66	0.72

**Table 3:** Segmentation metrics of HVDROPDB datasets with RetCam Images.

Evaluation Metrics	HVDROPDB-OD	HVDROPDB-BV	HVDROPDB-RIDGE
Accuracy	0.86	0.86	0.98
Precision	0.61	0.60	0.71
TPR	0.50	0.51	0.53
TNR	0.87	0.87	0.99
FPR	0.00	0.00	0.00
FNR	0.37	0.35	0.46
Dice Score	0.52	0.52	0.53

## Ethics Statements

Pictures acquired from premature babies participating in the hospital's screening program are utilized in a manner that maintains anonymity. As part of the procedure, the parents of these preterm infants provide written informed consent, permitting the utilization of the data for research and quality assurance purposes before the retinopathy of prematurity (ROP) screening. Institutional ethics committee approval was obtained on April 13 2020 (HVD/IEC/BHR/07/2020).

## CRediT authorship contribution statement

**Ranjana Agrawal:** Conceptualization, Data curation, Methodology, Writing – original draft. **Rahee Walambe:** Writing – review & editing. **Ketan Kotecha:** Supervision. **Anita Gaikwad:** Data curation. **Col. Madan Deshpande:** Supervision. **Sucheta Kulkarni:** Project administration, Data curation, Investigation, Validation.

## Data Availability

Research Data (Original data) (Mendeley Data)
